# Preceding Vehicle Detection and Tracking Adaptive to Illumination Variation in Night Traffic Scenes Based on Relevance Analysis

**DOI:** 10.3390/s140815325

**Published:** 2014-08-19

**Authors:** Junbin Guo, Jianqiang Wang, Xiaosong Guo, Chuanqiang Yu, Xiaoyan Sun

**Affiliations:** 1 Xi'an Institute of High-Tech, Xi'an 710025, China; E-Mails: gjb_202@163.com (J.G.); gjb202@sina.com (X.G.); fishychq@163.com (C.Y.); 2 State Key Laboratory of Automotive Safety and Energy, Tsinghua University, Beijing 100084, China; 3 Suzhou INVO Automotive Electronics Co., Ltd., Suzhou 215200, China; E-Mail: sunxiaoyantsj@126.com

**Keywords:** computer vision, driver assistance systems, preceding vehicle detection, taillight detection, relevance analysis, night traffic scenes

## Abstract

Preceding vehicle detection and tracking at nighttime are challenging problems due to the disturbance of other extraneous illuminant sources coexisting with the vehicle lights. To improve the detection accuracy and robustness of vehicle detection, a novel method for vehicle detection and tracking at nighttime is proposed in this paper. The characteristics of taillights in the gray level are applied to determine the lower boundary of the threshold for taillights segmentation, and the optimal threshold for taillight segmentation is calculated using the OTSU algorithm between the lower boundary and the highest grayscale of the region of interest. The candidate taillight pairs are extracted based on the similarity between left and right taillights, and the non-vehicle taillight pairs are removed based on the relevance analysis of vehicle location between frames. To reduce the false negative rate of vehicle detection, a vehicle tracking method based on taillights estimation is applied. The taillight spot candidate is sought in the region predicted by Kalman filtering, and the disturbed taillight is estimated based on the symmetry and location of the other taillight of the same vehicle. Vehicle tracking is completed after estimating its location according to the two taillight spots. The results of experiments on a vehicle platform indicate that the proposed method could detect vehicles quickly, correctly and robustly in the actual traffic environments with illumination variation.

## 1. Introduction

Statistics in the EU, USA and China indicate that the rear-end collisions are a principal cause of accidents on the highway, and the risk of the traffic accidents at night is greater than that in the daytime [1–3]. Thus, many researchers have attempted to use the rear-end collision warning systems to prevent these accidents. Preceding vehicle detection and tracking at night is one of the fundamental components for advanced driver assistance systems (ADAS). Over the past decade, a number of vehicle-mounted tracking and detection systems have been developed [4], which are based on active detection methods by the sensors such as radar [5] or laser [6]. Because of the advantages, such as abundant information, broad sensing range and low cost, the monocular-vision-based systems have been widely used in ADAS [7].

Due to the low contrast between vehicles and the background under badly illuminated conditions in nighttime road environments, the typical features applied to detect vehicles in the daytime, such as vehicle shadows, horizontal and vertical edges, and corners, cannot work well. The most significant feature of night preceding vehicles in dark environments is their red and bright taillights, and many studies of preceding vehicle detection at night have been completed by detecting the taillights [8–25].

In general, the taillights are among the brightest regions in the nighttime traffic videos. Grayscale or brightness threshold is a preferred method to segment the taillights from images [8,9]. The usual method is to establish heuristic rules for detection by setting a series of experiential thresholds [10–12]. Machine learning techniques have also been applied to classify the taillights [13], including back-propagation neural network [14], Bayes factor [15], support vector machine [16,17], and Real Adaboost algorithm [18,19]. Considering the color of taillights, the red-color filter was employed to extract the true taillights and remove the non-vehicle light sources. The red-color light regions that include actual taillights are segmented into many different color spaces with widely varying parameters. The differences of the red value and the blue value [20] and normalized red intensities [21] have been utilized to extract the red-color pixels in the RGB color space. O'Malley [22] determined the threshold to extract taillights by census of more than 300 tail and brake lamps in the HSV color space [23]. Taillights extraction could also be accomplished using the Cr component of the YCrCb color space [24]. However, all the thresholds are subjective, and the ability to be adaptive to illumination variation is poor.

After extracting the taillights from images, the similarities between left and right taillights' height, size, shape, and color are utilized to pair the actual taillights [25]. However, many other illuminant sources coexist with the vehicle lights in nighttime road environments, such as street lamps, traffic lights, and road reflector plates on the ground. Their grayscale may be higher than that of a vehicle light. Therefore, these non-vehicle illuminant sources could cause many difficulties for detecting the actual vehicles in nighttime road scenes. Meanwhile, these vehicle detection methods do not consider the correlation of the same vehicle in the preceding and current frame, and their detection rates and robustness are poor.

Vehicle tracking can be employed to improve the detection rates and robustness of vehicle detection systems. Existing vehicle tracking methods could be divided into vehicle tracking based on template matching [26–28] and vehicle tracking based on a motion model [29–31]. The former method detects the vehicles in the previous frames, and the potential vehicle in the current frame is matched with the tracking template according to specific rules. Either a single taillight [32] or taillight pair [33] could be used as the tracking object by selecting different characteristics. The disadvantages of these methods are that the uniform template is difficult to build and the computational cost is high. The latter method estimates the vehicle location in the current frame according to motion model. The typical methods are the Kalman filtering method [34–37] and particle filtering tracking method [38]. It uses the previously estimated state and the current measurement to estimate the current state. The estimate value can be used to compensate for the current unavailable detection. The current state is estimated by combining the previously estimated state and the current measurement. However, these methods cannot deal with the false pairing from different vehicles, and the tracking process will increase the computational complexity. The tracking method based on template matching combined with Kalman filtering is employed to track target vehicles [29]. When the vehicle is not detected based on the matching characteristics, the estimated state predicted by Kalman filtering would be regarded as the vehicle position in the current frame. This tracking method could reduce the false negative rate of vehicle detection effectively, but it is ineffective to reduce the false detection rate. Considering that these tracking methods based on state estimation depend on the global vehicle detection results, there have limitations, including high false negative rate in complex road environments, high computational costs, and poor real-time performance.

From the above review of related research, we observe that three main issues remain to be resolved. First, as the intensity and color of the taillight in an image is dependent on the illumination condition and sensor characteristics, existing taillight segmentation methods are weak to be adaptive to illumination variation. Second, the true taillight pair may be eliminated because the correlation of the same vehicle in the preceding and current frames may be ignored. Finally, existing night vehicle detection methods do not work or cannot locate the true vehicle accurately when one taillight is disturbed by other illuminant sources.

To promote correctness and robustness, a novel monocular-vision-based night vehicle detection and tracking method, which relies on taillight characteristics, is presented in this paper. Compared with the existing methods, the main contributions of the proposed method include the following aspects: (i) A taillight segmentation method based on an improved OTSU algorithm is proposed to promote the ability to be adaptive to illumination condition variations. (ii) After pairing the spots according to certain characteristics such as size, height, and symmetry, the incorrect vehicles are eliminated by the correlation with the actual vehicle in the previous frame. This can reduce the false detection rate and improve the detection rate. (iii) A vehicle-tracking method is proposed based on relevance analysis and taillight estimation, which can track the true vehicle accurately when one taillight is disturbed by other illuminant sources. Experimental results demonstrate that the presented method could obtain high detection rate, low false positive and false negative rates, and high robustness at different lighting conditions and road environments.

The remaining sections are organized as follows: Section 2 introduces the taillight segmentation method adaptive to illumination condition variation. In Section 3, we present the vehicle detection algorithm based on taillight pairing and relevance analysis. Section 4 presents the vehicle tracking algorithm based on relevance analysis and taillight estimation. The experimental results and system performance are presented in Section 5. Finally, conclusions of this research are described in Section 6.

## 2. Taillights Segmentation

The intensity of the taillights in an image depends on the distance from the target to the camera. In addition, the appearance of taillights in the captured video is highly dependent on the camera settings and sensor characteristics. Therefore, the taillight segmentation results cannot accurately be replicated or verified, and may vary with different camera hardware. Compared with the whole image, the size of one taillight is so bright and small that the ratio of taillights' grayscale would be very low in the whole histogram. Thus, the ideal threshold could not be derived by analyzing the histogram of the whole image. It is appropriate to divide an image into several sub-blocks, and the threshold in each sub-block is different. If these sub-blocks are uniformly distributed in an image, the segmentation result would be disappointing because of the block effect. To promote the proportion of taillight in the pixels of the background, Wang [39] and Sun [40] proposed to find the threshold for taillight segmentation between an empirical value and the highest grayscale based on the OTSU algorithm. Because the work is completed in the cumulative intensity histogram of 15 consecutive frames and the lower boundary of the threshold is set based on experience, it is complex and its adaptability to illumination variation is limited.

**Figure 1.**
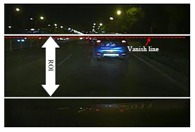
Region of Interest (ROI) of a typical image of a nighttime traffic scene.

To segment the taillights exactly and robustly, a taillight segmentation method adaptive to illumination condition variation based on the improved OTSU algorithm is proposed in this section. First, counting the average number of higher grayscales is counted and the lower boundary of the threshold is determined after analyzing the histogram of the whole image. Secondly, the original optimal threshold is calculated by the OTSU method in the interval between the lower boundary and the highest grayscale of the whole image, and the image is divided into several subblocks by this threshold. All the operations are accomplished in the region of interest (ROI), which is restricted by the vanishing line and the corresponding line of the head of the host vehicle. A typical image of a nighttime traffic scene is shown in Figure 1, where is limited by the white lines and the red dotted line is the vanishing line. The experiments demonstrate the robustness and satisfactory results to illumination condition variation.

**Figure 2.**
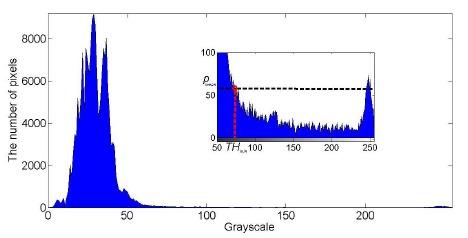
Histogram of the ROI in a typical image of a nighttime traffic scene.

The corresponding histogram of the ROI in Figure 1 is shown in Figure 2, where the histogram from 80 to 255 is magnified as a sub-image. As shown in Figure 2, the histogram has two peaks; the higher peak with the smaller grayscale represents the pixels of background while the lower peak with bigger grayscale represents the brighter pixels that include taillights. If each peak is fitted with one gauss curve, the global threshold would be the intersection of the two curves.

In order to reduce the computation complexity, the lower boundary of the threshold could be estimated according to the distribution of the whole image histogram. In one original image, *G*_max_ denotes the maximum grayscale in the whole image, and *p*(*i*) represents the proportion of pixels with a grayscale of *i*. Then, as shown in Figure 2, the average of proportion *p_mean_* between *G*_max_ − *δ* and *G*_max_ could be calculated as follows:

(1) $$p_{\mathrm{mean}}=\frac{1}{\delta }\sum_{G_{\max}-\delta }^{G_{\max}}p(i)$$

Where *δ* is determined based on experience, which is a value between 10 and 20. From the gray value with the maximum proportion to the maximum grayscale, the first grayscale whose proportion is smaller than *p_mean_* is the lower boundary *Th*_min_. The original optimal threshold could be calculated by the OTSU method between *Th*_min_ and *G*_max_ The brighter pixels could be extracted from the background by the original optimal threshold. One block that is bigger than the possible size of one taillight could be a taillight adjoining some brighter lights. After counting each block's size, the block whose size is bigger than the possible size would be segmented by the average gray of all pixels in the block again. Thus, the real taillight could be extracted from the background completely.

**Figure 3.**
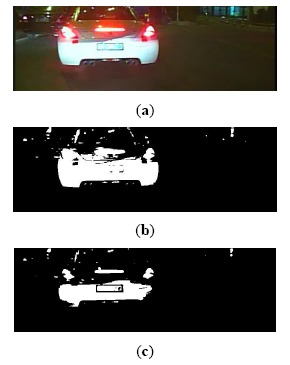
Result of taillights segmentation: (**a**) Original image; (**b**) Result of taillights segmentation by the common OTSU method; (**c**) Result of taillights segmentation by the proposed method.

The result of taillight segmentation by the proposed method is shown in Figure 3. It can be observed that the real taillight is adjoined with the brighter reflector in the back of the car in the segmentation image by the common OTSU method, as shown in Figure 3b. However, the proposed method could extract the taillights entirely, as shown in Figure 3c. Hence, the proposed method could extract the real taillights from the background effectively.

## 3. Vehicle Detection Based on Taillight Pairing and Relevance Analysis

In this section, we provide a vehicle detection method based on taillight pairing and relevance analysis between frames. First, the potential vehicle is detected using the global rule-based algorithm. The pairing algorithms apply heuristics, such as area ratio, symmetry, and bounding box aspect ratio. Because the rules of pairing taillights are usually limited by fixed thresholds and removing the vehicle candidate that possesses the lesser similarity of two spots, the global rule-based vehicle detection may obtain some incorrect taillight pairs in some real traffic scenes. To promote the correctness of vehicle detection, the relevance of location is used to remove the non-taillight pairs.

In the night scenes, the preceding vehicles are primarily visible by their red-color rear-facing taillights. Vehicle equipment differs in appearance, style, and heights, but all the taillights of different vehicles are within specified limits for color and brightness. In addition, the left and right taillights in the same vehicle must be placed symmetrically and in pairs with the same shape and size. Thus, we could pair the real taillights according to these regulations by deriving image processing system parameters from them. However, there could be some non-taillight pairs since we pared spots only using the similarity and these pairs should be removed. According to the temporal continuity of video data, the motion between the adjacent frames is very slight. Therefore, the wrong pairs near to the detected vehicle in the previous frame could be removed by comparing the motion from the location in the previous frame to the current location. In addition, the remaining wrong pairs that overlap with others could be removed by comparing the similarity and symmetry of the left and right spots.

### 3.1. Taillight Pairing Based on Similarity Analysis

Because the two taillights on the same vehicle are placed symmetrically and in pairs, they have the same shape and size. Ignoring installation errors and vehicle vibration, the size, width, and height of the two real taillights' spots are very near. With typical vehicles such as passenger cars, recreational vehicles, jeeps, and small freight cars, the vehicle width is about 170 cm, and the aspect ratio (width/height) of vehicles is usually approximate to 2.0. Thus, the brighter spots could be paired according to the regulations mentioned above.

Initially, we define the candidate spot as *B_i_*(*x_i_,y_i_,A_i_*), where *x_i_* and *y_i_* are the coordinates of the centroid, and *A_i_* is the area. The location of the bounding boxes of region *B_i_* used in the spatial clustering process are their top, bottom, left and right coordinates, and they are denoted as *T*(*B_i_*), *B*(*B_i_*), *L*(*B_i_*), and *R*(*B_i_*), respectively. Likewise, the parameters of *B_i_* could be defined. If *B_i_* and *B_j_* are the real taillights' spots on the same vehicle, they should satisfy the restraints of similarity and symmetry as follows:

(1) Initially, because the heights of two taillights on the same vehicle are very near, their longitudinal coordinates should satisfy the restrictive condition as shown below:
  (2) $$\left|y_{i}-y_{j}\right|<\Delta h$$
  where Δ*h* is a very small tolerant value caused by the taillight segmentation and centroid detection.
(2) Secondly, as the area of lights theoretically should not vary too much either, we define another condition for removing pairs that have a large difference between the areas of both lights:
  (3) $$\frac{\max(A_{i},A_{j})-\min(A_{i},A_{j})}{\min(A_{i},A_{j})}<\Delta A$$
  where Δ*A* is a tolerant value for the difference of the two taillights' area.
(3) The aspect ratio (width/height) of a candidate pair could be obtained according to the location of the bounding boxes of two spots in a pair as follows:
  (4) $$R=\frac{\max(R(B_{i}),R(B_{j}))-\min(L(B_{i}),L(B_{j}))}{\max(B(B_{i}),B(B_{j}))-\min(T(B_{i}),T(B_{j}))}$$
  As a real taillight pair has its own shape, the aspect ratio must be in an interval:
  (5) $$\Delta R_{\min}\leq R\leq \Delta R_{\max}$$
(4) Considering the aspect ratio (width/height) of a vehicle is approximate to 2.0, the vehicle projection line on the ground could be estimated by the width and location of the taillight pair. Therefore, the width and distance between the ego-vehicle and the preceding vehicle can be calculated by the relationship from image to real scene [41]. Thus, the width condition could be defined to remove the pairs with inappropriate width:
  (6) $$\Delta W_{\min}\leq W_{V}\leq \Delta W_{\max}$$
(5) Because the taillights were placed symmetrically on the vehicle, the symmetry of the two spots in a pair could be applied to remove some non-taillight pairs. Supposing *B_i_* and *B_j_* are two spots in a pair, and *B_i_′* is the mirror image of *B_i_*. Then the symmetry of the two spots *B_i_* and *B_j_* could be represented by the similarity of *B_i_′* and *B_j_*. Counting the number of pixels overlapped on *B_i_′* and *B_j_*, which is denoted as *A_s_*, then the similarity could be represented as follows:
  (7) $$S=\frac{A_{s}}{\max(A_{i},A_{j})}$$
  By defining the symmetry threshold as Δ*S*, the symmetry of the two spots in a pair must satisfy the condition:
  (8) S≤ΔS

**Figure 4.**
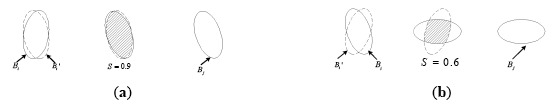
Examples of symmetry of two spots: (**a**) Symmetry of taillights in the same; (**b**) Symmetry of taillights in different.

The typical examples are shown in Figure 4, and the values of two spots on the same vehicle and different vehicles are calculated. From Figure 4 we can observe that the symmetry of taillight spots in the same vehicle is higher than that of taillight spots in the others.

**Figure 5.**
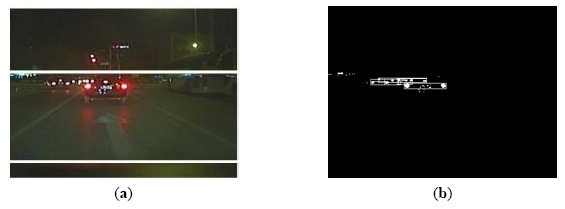
Result of taillight pairing based on the similarity analysis: (**a**) Original image; (**b**) Result of taillight pairing.

Because a difference exists in the taillight height, an error of the vehicle projection line on the ground occurs by estimating according to the aspect ratio and bounding boxes of the taillight pair. Due to the existing vibration of the moving vehicle, the accuracy of the estimated vehicle width is not very high. Thus, the estimated vehicle width may be limited in a wide range, and *W* could vary between 1.2 and 2.2 m by experience. Meanwhile, the size and shape may vary with the different distance between the ego-vehicle and the preceding vehicle, but the size and shape of one taillight changes the same as that of the other. Therefore, the other thresholds about similarity could be limited strictly. They could be determined by experience, *i.e.*, Δ*h* is admitted between 1 and 3, Δ*A* is between 1 and 2, Δ*S* is about 0.5, and *R* is admitted to vary from 3 to 15. The original image and the result of taillight pairing based on the similarity of spots are shown in Figure 5.

### 3.2. Removing the Non-Vehicle Taillight Pair Based on Relevance Analysis

As mentioned above, the candidate vehicles that are extracted based on the similarity analysis inevitably include the non-taillight pairs. Thus, the real taillight pair might be removed if the overlapped candidate vehicles are compared absolutely by the similarity of two taillight spots. In this part, we provide a novel method to remove the non-vehicle taillight pair based on relevance analysis.

Considering the temporal continuity of preceding vehicle movement, taillight pairs of the same vehicle have the minimum distance to each other between the current and previous frame changes [42]. Therefore, the non-taillight pairs overlapped with the vehicle location in the previous frame could be removed by comparing the degree of overlapping. The pair with the highest degree of overlapping is the true taillight pair. The overlapped pairs that are not overlapping the vehicle detected in the previous frame could be verified by comparing the symmetry of the two spots in a pair. The higher is the symmetry, the more possible it is a real taillight pair. Based on these considerations, the non-taillight pairs could be removed.

Suppose that the candidate vehicle is represented by one taillight pair as *V_m_*, and the corresponding spots are *B_i_* and *B_j_*. Then, the four sides of the rectangle surrounding the vehicle are defined as *L*(*V_m_*), *R*(*V_m_*), *T*(*V_m_*) and *B*(*V_m_*), respectively:

(9) $$L(V_{m})=\min(x_{i},x_{j})$$

(10) $$R(V_{m})=\max(x_{i},x_{j})$$

(11) $$B(V_{m})=\left(y_{i}+y_{j}+\left|x_{i}-x_{j}\right|\right)/2$$

(12) $$T(V_{m})=\left(y_{i}+y_{j}-\left|x_{i}-x_{j}\right|\right)/2$$

Likewise, the four sides of the candidate vehicle *V_n_* are defined as *L*(*V_n_*), *R*(*V_n_*), *T*(*V_n_*) and *B*(*V_n_*), respectively. Then the horizontal distance Δ*d_x_* and vertical distance Δ*d_y_* between the two candidate vehicles are expressed as

(13) $$\Delta d_{x}=\max\left(L(V_{m}),L(V_{n})\right)-\min\left(R(V_{m}),R(V_{n})\right)$$

(14) $$\Delta d_{y}=\max\left(T(V_{m}),T(V_{n})\right)-\min\left(B(V_{m}),B(V_{n})\right)$$

**Figure 6.**
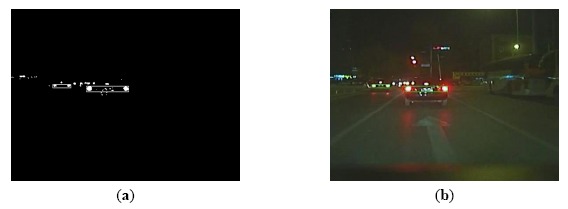
Results of vehicle detection based on taillight pairing and relevance analysis: (**a**) Vehicle detection result in binary image; (**b**) Vehicle detection result in color image.

Through the above two equations, it can be observed that both Δ*d_x_* and Δ*d_y_* are negative when the two candidate vehicles are overlapped. Because the vehicles could not be overlapped in the image, there must be non-taillight pairs that should be removed when an overlapping is detected. Figure 6 shows the detected vehicle from the overlapped vehicle candidates after the non-vehicle pairs are removed.

(1) Initially, by analyzing the location of each candidate vehicle *V_i_* with the detected vehicle *V_d_* in the previous frame, the overlapped area in the image could be counted, denoted as *A_C_*. The true vehicle should satisfy the following conditions:
  (15) $$A_{C}/A(V_{i})\geq \Delta A_{C}$$
  (16) $$A_{C}/A(V_{d})\geq \Delta A_{C}$$
  where *A*(*V_i_*) is the area of the rectangle surrounding the vehicle *V_i_* and Δ*A_C_* is a threshold for degree of overlapping. The candidate vehicles that overlap with the detected vehicle *V_d_* and do not satisfy the above two restraints could be removed. If several candidate vehicles exist that satisfy the above two restraints, the movement between the candidate vehicle and the detected vehicle in the previous frame is calculated as follows:
  (17) $$S=\left|L(V_{i})-L(V_{d})\right|+\left|R(V_{i})-R(V_{d})\right|$$
  As the movement is very little between the sequential frames, the candidate vehicle with the least value of *S* is kept, and the other candidate vehicles overlapping with *V_d_* could be removed. Repeating the above work, the real vehicles corresponding to the detected vehicles in the previous frame are extracted.
(2) Secondly, to other candidate vehicles, non-taillight pairs exist when one candidate vehicle is overlaps with another one. By comparing the shape difference of two spots in a pair, using parameters such as size, height, and symmetry, the candidate vehicle with the highest similarity is the most likely to be a real vehicle and the candidate vehicles overlapping with it could be removed. The above work is repeated until no vehicle overlaps with another.

For the remaining detected vehicles, only the vehicles that appeared in five sequential frames were considered one true vehicle to reduce the effect of temporary disturbances. To improve the detection rate and robustness, vehicle tracking is implemented based on these true vehicles, as illustrated in the next section.

## 4. Vehicle Tracking

When the taillight is destroyed by other illuminant sources or reflectors, vehicle detection based on taillight pairing will fail. To interpolate the correct position of the vehicle in this situation, we provide a new vehicle tracking method in this section based on relevance analysis and taillight estimation in this section. The vehicle tracking based on taillight estimation can be summarized as follows: the taillight spots are extracted by analyzing relevance of location, and the vehicle is estimated by the extracted taillight spots.

### 4.1. Taillight Spot Extraction

Considering the short time between the sequential frames, the features of real taillights, such as location, shape, and size, do not vary much. First of all, the location of vehicle could be estimated by the Kalman filtering method, and then the taillight spot is sought around the location. The relationship between sequential frames could be applied to find the real taillight spots.

#### 4.1.1. Taillight Location Predication Based on Kalman Filtering

In this paper, the Kalman filter method, a least-squares estimation of linear movement, is used to track the targets. In the previous step, vehicles were detected and the coordinates of their center points of project line on the ground and speeds in the two coordinate directions were used as the parameters of the state vector, which are denoted by (*x,y*) and (*ẋ ẏ*), respectively. Then the state vector *X* is denoted by:

(18) $$X={\left(x,y,ẋ,ẏ\right)}^{T}$$

The prediction of the state vector *X̂*_*k*+1_ could be estimated by the state transition equation:

(19) X(k)=A(k|k−1)X(k−1)+w(k−1)

The measurement equation is expressed as follows:

(20) Z(k)=C(k)X(k)+r(k)

where *A*(*k*|*k* − 1) and *C*(*k*) are the state transition and measurement matrix, respectively. They can be defined as follows:

(21) $$A(k|k-1)=\left[\begin{array}{cccc} 1 & 0 & t & 0\\ 0 & 1 & 0 & t\\ 0 & 0 & 1 & 0\\ 0 & 0 & 0 & 1 \end{array}\right]$$

(22) $$C(k)=\left[\begin{array}{cccc} 1 & 0 & 0 & 0\\ 0 & 1 & 0 & 0 \end{array}\right]$$

where *w*(*k*) and *r*(*k*) are assumed to be independent, zero-means, white Gaussian noise with their own covariance matrices *W_k_* and *R_k_*, and *t* is the sampling time for one frame. After estimating the location of one vehicle, its taillight spot is sought around the estimated location.

#### 4.1.2. Taillight Spot Extraction Based on Relevance Analysis

Because the sampling time of the image is very short, the taillight centroid moves very slightly. Normally, the centroid of the real taillight spot is nearest to that predicted by Kalman filtering. However, the real taillight spot may adjoin with other bright spots, such as other lights, and reflectors, thus the real taillight spot would be missed if the spot is only sought based on the distance between the two centroids.

The area of the taillight in an image varies with the distance between the ego-vehicle and the preceding vehicle. When the preceding vehicle is far from the ego-vehicle, the area of the taillight is small and the movement of the taillight centroid is very slight. Meanwhile, the movement of the taillight centroid is larger when the preceding vehicle is near to the ego-vehicle but the area of the taillight is also larger. Thus, there must be some pixels of real taillight spots that appear in a small neighborhood *N* of the predicted taillight centroid. Letting *B_i,k_* be the spot of the real taillight denoted as *L_i_* at time *k*, and *N* be the neighborhood of its centroid, the taillight spot at time *k* + 1 must satisfy the expression:

(23) $$B_{i,k+1}\in \left\{B_{j,k+1}|B_{j,k+1}\cap N\neq \emptyset \right\}\;j=1,2,3,\ldots \ldots$$

The motion of the preceding vehicle relative to the ego-vehicle is a concerned in ADAS. The relative motion includes the longitudinal, lateral, and composite movements. The longitudinal movement occurs when the ego-vehicle is following, near to or far from the preceding vehicle. As mentioned above, some pixels of the real taillight must appear in a small neighborhood of the centroid of the taillight in the previous frame. The lateral movement occurs when the preceding vehicle cuts into or out of the lane. The movement of the left and right taillights on the preceding vehicle is the same. Based on the above characteristics, a relationship of taillight location and movement exists between the current and previous frames. Therefore, the taillight spot could be found around its predicted taillight centroid according to these relationships.

When the taillights are segmented completely and exactly, the spot nearest to its predicted taillight centroid is the real taillight. Thus, we can choose the spot including the nearest pixel to the centroid as the real taillight spot, and the area change is applied to verify the spot. If one of the two taillight spots is not found for various reasons, the movement relationship of the two taillights is considered to determine the taillight spots.

Generally, the distance is represented by the distance between one spot centroid and the taillight spot centroid in the previous frame. This may lead to missing the real taillight spot when it is adjoined with other lights or reflectors. Therefore, in this paper, the distance of one spot is defined by the minimal distance of any pixel in one spot to the predicted taillight centroid. In order to reduce the computation, the spot including the pixels in a neighborhood of the predicted taillight centroid is the candidate. Supposing that the location of one taillight in the previous frame is *L*(*x_L_,y_L_,A_L_*), then the predicted centroid of this taillight by Kalman filtering is (*x̂_L_*,*ŷ_L_*), and the distance between the spot and the predicted taillight centroid is defined as follows:

(24) $$d(B_{m},L)=\underset{i}{\min}\sqrt{{\left(x_{i}-{\hat{x}}_{L}\right)}^{2}+{\left(y_{i}-{ŷ}_{L}\right)}^{2}}$$

where (*x_i_,y_i_*) is any pixels belonging to both *B_m_* and *N_L_*, (*x_i_,y_i_*)∈*B*∩*N_L_*, where *N_L_* is a small neighborhood about the predicted taillight centroid. The possibility of one spot to be a taillight is defined as follows:

(25) $$P(B_{m},L)=\frac{1}{1+d(B_{m},L)}$$

As mentioned above, the spot with the maximal possibility is selected as the taillight spot. In order to ensure the correctness, the spots are verified by the change of area when they differ. The confidence of the spot is defined as follows:

(26) $$C(B_{m},L)=1-\frac{\left|A_{B_{m}}-A_{L}\right|}{A_{L}}$$

The higher the confidence, the more possible the spot is the real taillight is. The spot whose confidence is higher than a threshold could be selected as the real taillight spot. The real taillight spot is determined as follows:

(1) First, the distance of each spot to the predicted centroid is analysed. After analyzing the distance and verifying the confidence of the area change, the appropriate spots corresponding to the left and right taillights are found. Then, the vehicle in the image could be determined by the location of the two spots. If the spots corresponding to the left and right taillights are the same ones, the left and right taillights are adjoined with the reflector in the back of the car. Then the vehicle location in the image could be determined by the longitudinal coordinate and the left and right sides of the spot.
(2) Second, only one of the two appropriate spots is found by analyzing the distance and area. Another taillight spot may be selected by ignoring the area verification in a larger neighborhood of the predicted centroid. If the appropriate spot is found, the vehicle in image could be determined by the location of the two spots. When it is unsuccessful, the actual taillight may be out of the range predicted by the Kalman filter. The centroid predicted by the Kalman filtering is corrected according to the spot movement found by analyzing the distance and area. Generally, supposing that the spot corresponding to taillight *L_m_* found by analyzing the distance and area is *B_k_*(*x_k_,y_k_*), then the predicted centroid is (*x̂_m_*,*ŷ_m_*), and the movement of the taillight could be estimated by the following equations:
  (27) $$\Delta x=x_{k}-{\hat{x}}_{m}$$
  (28) $$\Delta y=y_{k}-{ŷ}_{m}$$
  The predicted location of the other taillight *L_n_* is revised as (*x̂_m_* + Δ*x*, *ŷ_m_* + Δ*y*). Then the corresponding spot is searched in the neighborhood of the revised location by analyzing the distance. The location of vehicle is determined by the taillight estimation.
(3) Finally, the location of the vehicle is predicted by the Kalman filtering when no appropriate spot is found by the above-mentioned method. In addition, the taillight parameters are revised by the prediction of Kalman filtering. Vehicle tracking is stopped when an appropriate spot has not been found in five sequential frames.

### 4.2. Vehicle Location Estimation Based on Extracted Taillights

From the above-mentioned conclusions, the vehicle location could be estimated in the image. The vehicle location is predicted by Kalman filtering when either the left or right appropriate spot is not found. Vehicle tracking would be stopped when an appropriate spot is not found in five sequential frames. If one of the left and right taillight spots is found, the vehicle location could be estimated according to these taillight spots. For each taillight pair, supposing that *B_i_*(*x_i_,y_i_*) is the left one and *B_j_*(*x_j_,y_j_*) is the right one, then the vehicle location estimation algorithm is presented as follows:

(1) If *B_i_* and *B_j_* are on the same vehicle, the vehicle location is estimated by the left and right side of the spot and vehicle location in the previous frame. Letting the four sides of rectangle around vehicle *V_m_*_,_*_k_* at time *k* be *T*(*V_m_*_,_*_k_*), *B*(*V_m_*_,_*_k_*), *L*(*V_m_*_,_*_k_*), and *R*(*V_m_*_,_*_k_*), respectively; then the location at time *k* + 1 can be estimated by the following equations:
  (29) $$T(V_{m,k+1})=T(V_{m,k})$$
  (30) $$B(V_{m,k+1})=B(V_{m,k})$$
  (31) $$L(V_{m,k+1})=L(B_{i})$$
  (32) $$R(V_{m,k+1})=R(B_{i})$$
(2) If *B_i_* and *B_j_* are not on the same vehicle, the two spots, or one of them, is the real spot of the taillight. The vehicle location should be estimated by the real shape of the taillights. When the two spots are the real spots of the taillight, the taillights are segmented exactly. Then the location at time *k* + 1 could be estimated by the bounding boxes of the two spots.
  (33) $$L(V_{m,k+1})=\min\left(L(B_{i}),L(B_{j})\right)$$
  (34) $$R(V_{m,k+1})=\max\left(R(B_{i}),R(B_{j})\right)$$
  (35) $$T(V_{m,k+1})=\left(y_{i}+y_{j}-R(V_{m,k+1})+L(V_{m,k+1})\right)/2$$
  (36) $$B(V_{m,k+1})=\left(y_{i}+y_{j}+R(V_{m,k+1})-L(V_{m,k+1})\right)/2$$

When one of the taillights is disturbed by others, the disturbed taillight shape could be estimated from that of the other one. Supposing that *L_m_*(*x_m_,y_m_*) and *L_n_*(*x_n_,y_n_*) are the left and right taillight spots, respectively, and *B_i_* is the spot segmented exactly while *B_j_* is not. The variations of three kinds of distances, the farthest and nearest distance between the pixels of two spots and the center distance, are calculated:

(37) $$\Delta d_{C}=\left|\frac{x_{i}-x_{j}}{x_{m}-x_{n}}-1\right|$$

(38) $$\Delta d_{n}=\left|\frac{L(B_{j})-R(B_{i})}{L(L_{n})-R(L_{m})}-1\right|$$

(39) $$\Delta d_{f}=\left|\frac{R(B_{j})-L(B_{i})}{R(L_{n})-L(L_{m})}-1\right|$$

The disturbed taillight is located based on the smallest one of these distance variations and the taillight segmented exactly. Supposing that only the left spot *B_i_*(*x_i_,y_i_*) in a pair is segmented exactly, and the variation of the nearest distance Δ*d_n_* is the smallest one, then the four sides of the bounding boxes of the right spot could be estimated by applying the following symmetry:

(40) $$x_{n}=L(B_{n})+R(B_{m})-x_{m}$$

(41) $$y_{n}=y_{n}$$

(42) $$T(B_{n})=T(B_{m})$$

(43) $$B(B_{n})=B(B_{m})$$

(44) $$L(B_{n})=L(B_{n})$$

(45) $$R(B_{n})=L(B_{n})+R(B_{m})-L(B_{m})$$

**Figure 7.**
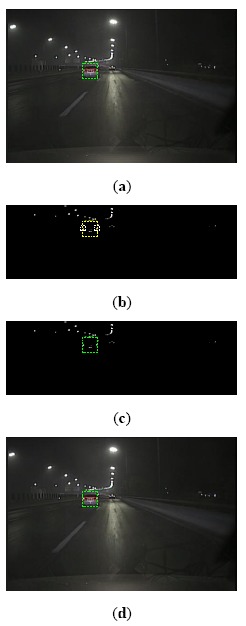
Procedure of vehicle tracking: (**a**) Vehicle detection result at time *k*; (**b**) Predicted location at time *k* + 1 based on Kalman filtering and taillight spot extraction; (**c**) Vehicle estimation based on extracted taillight; (**d**) Vehicle detection result at time *k* + 1.

The procedure and results of vehicle tracking are shown in Figure 7.

## 5. Experiment Evaluation

In this section, we provide several experimental studies to compare the performance of the presented algorithm with some classical studies existing in the literature. First, an experimental platform is constructed which could collect the original image of a real traffic scene. Then, the results of the proposed algorithm are compared with those of two other classical algorithms in several typical traffic scenes. Lastly, the detection ability of the three algorithms is given to demonstrate the correctness and robustness of the proposed algorithm.

### 5.1. Experimental Platform

**Figure 8.**
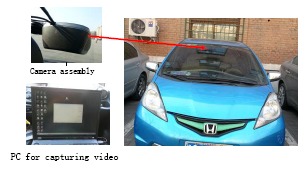
Platform for the vehicle experiment.

**Figure 9.**
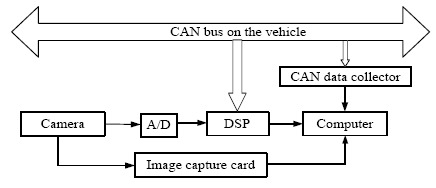
Structure of the experimental platform.

The platform is constructed for testing in an actual traffic environment. A CCD camera is mounted behind the driving mirror on the experimental passenger car (see Figure 8), and it is used to continuously monitor the traffic scenes when the car is moving. The camera is embedded in the camera assembly, and the assembly is firmly attached onto the front windshield firmly. A mobile computer equipped with an Intel Core i3 2.5 GHz processor and 2G RAM is used to capture the traffic video through an image capture card, and the captured image size is 768 × 576. The image from the camera is applied to detect and track the front vehicle based on digital signal processing (DSP), and the result could also be collected by the mobile computer. The current status of the ego-vehicle is collected from the CAN bus on the vehicle by the corresponding data collector. The structure of the experimental system is shown in Figure 9.

### 5.2. Experimental Result

In this section, we provide the results of several typical traffic scenes to demonstrate the superiority of the presented algorithm. The global rule-based algorithm, which is a simple and ordinary vehicle detection method, is the basis of many existing methods. To promote the detection rate, the tracking method is added onto the global rule-based algorithm. The tracking method based on feature matching combined with Kalman filtering is employed to track target vehicles by O'Malley [22,30], who verified the effectiveness of the tracking algorithm through a large number of experiments. The strategy presented by O'Malley *et al.* is summarized as follows: The target vehicle is tracking based on feature matching. If it fails to detect based on similar location, size, color, shape and symmetry, the position predicted by Kalman filtering is examined. The correlation coefficient of the candidate target and the corresponding region in the previous frame are calculated, and compared with the threshold value 0.85. In the following, compare the proposed algorithm with global rule-based algorithm and the Kalman tracking-based global algorithm proposed by O'Malley [30].

Excluding the superiority of taillight segmentation, the first advantage is that the proposed algorithm could avoid the false positive detections by utilizing the relevance of location between frames. In many actual traffic scenes, false positive detections usually occur when the real taillights are being paired with disturbing bright spots such as reflecting marks or taillights on another vehicle. Because the difference of the location between the adjacent frames is slight, the presented algorithm can remove any false pairs overlapped with the real taillight pair.

**Figure 10.**
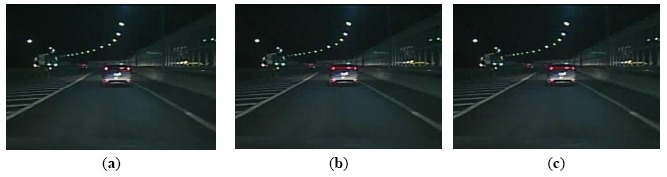
Comparison of vehicle detection results (case 1): (**a**) Result using the global rule-based algorithm; (**b**) Result using Kalman tracking-based global algorithm; (**c**) Result using the proposed algorithm.

Figure 10 shows the scene where a reflector of a street lamp is in the middle of a pair of taillights, and the reflector is more similar to the right taillight. The existing global rule-based algorithm could cause a false positive detection, as shown in Figure 10b. However, the proposed algorithm can eliminate the false pairing and keep the right taillight pair by considering the relationship of vehicle location between frames, as shown in Figure 10c.

**Figure 11.**
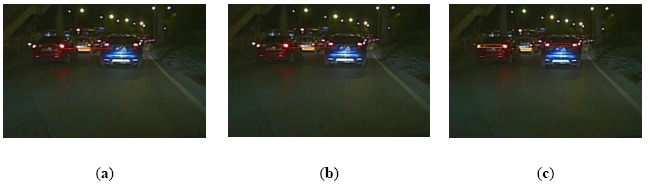
Comparison of vehicle detection results (case 2): (**a**) Result using the global rule-based algorithm; (**b**) Result using Kalman tracking-based global algorithm; (**c**) Result using the proposed algorithm.

Figure 11 shows a scene where there are several preceding vehicles at almost the same distances from the ego-vehicle. As the right taillight of the red car is more similar to the left taillight of the blue car than its own left taillight, the global rule-based algorithm would obtain a false positive result, as shown in Figure 11b. However, by utilizing the relevance of vehicle location between frames, the proposed algorithm could obtain the right detection result, as shown in Figure 11c. Overall, the above experiment results demonstrate that the proposed algorithm can reduce the false positive detection.

**Figure 12.**
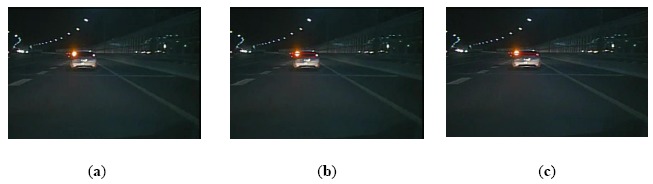
Comparison of vehicle detection results (case 3): (**a**) Result using the global rule-based algorithm; (**b**) Result using Kalman tracking-based global algorithm; (**c**) Result using the proposed algorithm.

Furthermore, another advantage of the proposed algorithm is the ability to track the vehicle successfully and correctly based on relevance analysis and taillight estimation. When one taillight was disturbed by others, such as reflector in the back of a vehicle or the headlight of an oncoming vehicle, the similarity between the left and right taillight spots is destroyed. Consequently, the global rule based algorithm cannot pair the spots of taillights. As the taillight spot is searched based on relevance analysis and the disturbed taillight is estimated according to similarity, the proposed algorithm can track the vehicle and reduce false negative detections, as shown in Figure 12, 13 through 14.

**Figure 13.**
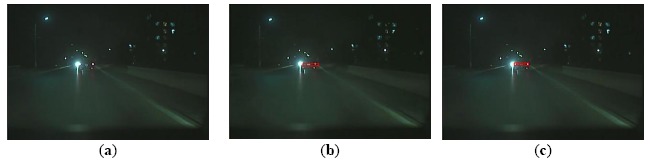
Comparison of vehicle detection results (case 4): (**a**) Result using the global rule-based algorithm; (**b**) Result using Kalman tracking-based global algorithm; (**c**) Result using the proposed algorithm.

**Figure 14.**
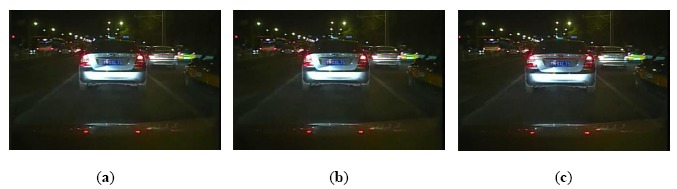
Comparison of vehicle detection results (case 5): (**a**) Result using the global rule-based algorithm; (**b**) Result using Kalman tracking-based global algorithm; (**c**) Result using the proposed algorithm.

Figure 12 shows a scene where the similarity of a pair of taillights is broken because the left turn light is flashing. The global rule-based algorithm would cause a false negative detection, as shown in Figure 12b. As the vehicle tracking strategy is based on taillight estimation, the disturbed taillight is located according to the undisturbed taillight. Thus, the proposed algorithm can obtain the taillight pair accurately, as shown in Figure 12c. Figure 13 describes a scene where one taillight is adhering to the headlight of the oncoming vehicle. The global rule-based algorithm could not pair the taillights, as shown in Figure 13b. Nevertheless, the proposed algorithm can obtain the correct vehicle location, as shown in Figure 13c. Figure 14 describes a scene where one taillight was adhering to the reflector in the back of the preceding vehicle, and the similar results are shown in Figure 14b and Figure 14c. Therefore, the experimental results shown in Figure 12, 13 through 14 indicate that the proposed algorithm can track a vehicle correctly and robustly, and thus the false negative detection rates can be reduced simultaneously.

### 5.3. System Performance

In order to test the validity of the proposed method, three typical traffic videos are applied for vehicle detection by the global rule-based algorithm, the Kalman tracking-based vehicle detection algorithm, and the proposed algorithm in this paper. The first video is captured at the Tsinghua University campus, without street lamps, and with only the headlights of oncoming vehicles for a short time and no other illuminants. The second video is captured on the highway, with street lamps, and contains the motions of the preceding vehicle cutting into or out of the lane of the ego-vehicle. The last video is captured on a city road, with street lamps, in which many other illuminant sources, including the street lamps, traffic lights, and reflectors on the ground or in the backs of cars, coexist with the vehicle lights.

To facilitate the analysis more clearly, all three videos documenting the three kinds of traffic scenes mentioned above are applied to vehicle detection by the proposed method. The videos are processed in the mobile computer equipped with an Intel Core i3 2.5 GHz processor and 2 G RAM. The experimental results demonstrate that the method is effective and takes no more than 33.65 ms to detect and track the preceding vehicle. The average processing time of each step and average total processing time per frame in the second video is shown in Table 1.

The proposed method is compared with the other two methods, and the performance parameters of detection rate, false positive rate, and false negative rate are shown in Table 2. The real vehicles in the video are labeled by hand, and the detected vehicle is compared with the labeled one in each frame. For simplicity, the global rule based algorithm is denoted by *M1*, the Kalman tracking-based vehicle detection algorithm proposed in [30] is denoted by *M2*, and the proposed method in the paper is denoted by *M3*. The labeled vehicle number is the sum of the amount of vehicles in each frame. In order to reduce the false positive detection, the comparison is completed only on the dangerous detected vehicles, which are those with a lateral distance to the ego-vehicle of less than 4 meters. Compared with the other two methods, the detection rate of the proposed method is higher, and the false negative rates and the false positive rates have been decreased.

## 6. Conclusions and Future Work

This paper proposed a robust vehicle detection and tracking method for different practical night traffic scenes using a single camera. The proposed method is able to improve the accuracy and robustness in a variety of traffic environments. The presented improved OTSU method can segment the taillight exactly in different night traffic scenes and can be adaptive to variations of illumination circumstance. Considering the location relevance of the vehicle in the current and previous frames, the proposed vehicle detection method can eliminate the non-taillight pairs quickly and correctly. The proposed vehicle tracking method based on taillight estimation and relevance analysis can track the vehicle correctly and robustly when the taillight is disturbed by other illuminant sources at night. The experimental results show that the proposed method significantly improves the detection rate while lowering false negative and false positive rates under different illumination circumstances and traffic scenes. The proposed method has correctly detected over 95% vehicles in night scenes with fine weather, and the false positive rates are less than 5%.

The proposed taillight pairing and relevance analysis approaches for vehicle detection utilize many heuristic fixed thresholds for determining the area ratio, symmetry, and bounding box aspect ratio of pairing taillights. Future work will include analyzing the sensitivity and influence of these parameters and finding the adaptive threshold for some important parameters.

## Figures and Tables

**Table 1.** Average processing time of the proposed method.

	**Step**	**Average Processing Time/ms**	**Average Total Processing Time per Frame/ms**
	
Vehicle detection and tracking based on relevance analysis	Threshold searching and image segmentation	6.490	14.101

	Block label	2.420	

	Taillight pairing and removing the non-vehicle taillight pair	0.941	

	Vehicle tracking	0.823	
	others	3.427	

**Table 2.** Comparison of the performance of different methods.

**Scene**	**Labeled Vehicle**	**Method**	**Detected Vehicle**	**Correct Detection**	**Detection Rate (%)**	**False Negative**	**False Negative Rate (%)**	**False Positive**	**False Positive Rate (%)**
Campus	2522	*M1*	2120	2086	82.7%	436	17.3%	34	1.4%
		*M2*	2174	2329	92.3%	193	7.7%	45	1.8%
		*M3*	2498	2492	98.8%	30	1.2%	6	0.3%
Highway	1598	*M1*	1189	1028	64.3%	570	35.7%	161	10.1%
		*M2*	1369	1364	85.4%	234	14.6%	165	10.3%
		*M3*	1548	1540	96.4%	58	3.6%	8	0.5%
City road	3633	*M1*	2851	2434	67.0%	1199	33.0%	417	11.48%
		*M2*	3254	2695	74.2%	938	25.8%	559	15.4%
		*M3*	3635	3484	95.9%	159	4.1%	151	4.2%
